# Factors associated with birth asphyxia among term singleton births at two referral hospitals in Northern Uganda: a cross sectional study

**DOI:** 10.1186/s12884-022-05095-y

**Published:** 2022-10-12

**Authors:** Elizabeth Ayebare, Claudia Hanson, Jolly Nankunda, Anna Hjelmstedt, Rebecca Nantanda, Wibke Jonas, James K. Tumwine, Grace Ndeezi

**Affiliations:** 1 Department of Nursing, School of Health Sciences, College of Health Sciences, Makerere University, Kampala, Uganda; 2 Department of Public Health Sciences, Karolinska Institutet, Stockholm, Sweden; 3 Department of Disease Control, London School of Hygiene and Tropical Medicine, London, UK; 4 Mulago Specialized Women’s & Neonatal Hospital, Kampala, Uganda; 5 Department of Paediatrics and Child Health, School of Medicine, College of Health Sciences, Makerere University, Kampala, Uganda; 6 Department of Women’s and Children’s Health, Karolinska Institutet, Stockholm, Sweden; 7 Makerere University Lung Institute, College of Health Sciences, Makerere University, Kampala, Uganda; 8 Department of Paediatrics and Child Health, School of Medicine, Kabale University, Kabale, Uganda

**Keywords:** Birth asphyxia, Socio-demographic characteristics, Teenagers, Maternal infections, Intrapartum complications, Northern Uganda

## Abstract

**Background:**

Birth asphyxia is one of the leading causes of neonatal mortality worldwide. In Uganda, it accounts for 28.9% of all neonatal deaths. With a view to inform policy and practice interventions to reduce adverse neonatal outcomes, we aimed to determine the prevalence and factors associated with birth asphyxia at two referral hospitals in Northern Uganda.

**Methods:**

This was a cross-sectional study, involving women who gave birth at two referral hospitals. Women in labour were consecutively enrolled by the research assistants, who also attended the births and determined Apgar scores. Data on socio-demographic characteristics, pregnancy history and care during labour, were obtained using a structured questionnaire. Participants were tested for; i) malaria (peripheral and placental blood samples), ii) syphilis, iii) white blood cell counts (WBC), and iv) haemoglobin levels. The prevalence of birth asphyxia was determined as the number of newborns with Apgar scores < 7 at 5 min out of the total population of study participants. Factors independently associated with birth asphyxia were determined using multivariable logistic regression analysis and a *p-value* < 0.05 was considered statistically significant.

**Results:**

A total of 2,930 mother-newborn pairs were included, and the prevalence of birth asphyxia was 154 [5.3% (95% confidence interval: 4.5- 6.1)]. Factors associated with birth asphyxia were; maternal age ≤ 19 years [adjusted odds ratio (aOR) 1.92 (1.27–2.91)], syphilis infection [aOR 2.45(1.08–5.57)], and a high white blood cell count [aOR 2.26 (1.26–4.06)], while employment [aOR 0.43 (0.22–0.83)] was protective. Additionally, referral [aOR1.75 (1.10–2.79)], induction/augmentation of labour [aOR 2.70 (1.62–4.50)], prolonged labour [aOR 1.88 (1.25–2.83)], obstructed labour [aOR 3.40 (1.70–6.83)], malpresentation/ malposition [aOR 3.00 (1.44–6.27)] and assisted vaginal delivery [aOR 5.54 (2.30–13.30)] were associated with birth asphyxia. Male newborns [aOR 1.92 (1.28–2.88)] and those with a low birth weight [aOR 2.20 (1.07–4.50)], were also more likely to develop birth asphyxia.

**Conclusion:**

The prevalence of birth asphyxia was 5.3%. In addition to the known intrapartum complications, teenage motherhood, syphilis and a raised white blood cell count were associated with birth asphyxia. This indicates that for sustained reduction of birth asphyxia, appropriate management of maternal infections and improved intrapartum quality of care are essential.

## Background

Birth asphyxia is defined by the World Health Organization (WHO), as failure to initiate and sustain breathing at birth [1]. It may also be defined by a five-minute Apgar score of less than 7 [2]. Birth asphyxia is a major cause of mortality among newborns, and accounts for 24% of the world’s neonatal deaths [3]. In Uganda, neonatal mortality has remained high at 27/1000 live births over the last decade [4], and birth asphyxia was responsible for 28.6% of these deaths [5]. In 2020/2021, the maternal and perinatal death surveillance and response report for Uganda showed that, 48% of all perinatal deaths were attributed to birth asphyxia [6]. Furthermore, infants who have experienced birth asphyxia, may suffer long-term neurological impairment, thus affecting their quality of life [7]. Studies report that children who had asphyxia at birth, have a significant risk for neurodevelopmental disability including; derangements in social behaviors, language and gross and fine motor skills [8, 9].

Interventions to address newborn deaths around the time of birth include: i) promotion of maternal birth preparedness, ii) skilled attendance at birth, iii) essential newborn care, iv) basic and comprehensive obstetric care, and neonatal resuscitation [10, 11]. Although Uganda has adopted this package as part of the guidelines for provision of maternity care services, the rate of newborn death is still high. This implies that the country needs more targeted interventions, in order to achieve the sustainable development goal target of reducing neonatal mortality, to less than 12 deaths per 1000 live births by 2030 [12]. These interventions will need to be designed to explicitly address local factors contributing to birth asphyxia [10].

The literature describes several factors associated with birth asphyxia as an interaction between predisposing factors, infections and birth complications [13]. While the main mechanisms in the development of birth asphyxia should be similar in all settings, there is a possible contribution of socio-demographic factors in this complex phenomenon. Socio-demographic factors such as parental education, socio-economic status, occupation and marital status have been associated with birth asphyxia [14–16]. These factors increase the risk of birth asphyxia since they affect a woman’s wellbeing during pregnancy and her access to good intrapartum care. In many low-income countries, delay to access emergency obstetric and newborn care contributes to the high rates of stillbirths and neonatal deaths [17], yet less is known in a post conflict setting such as Northern Uganda. Maternal infections around the time of birth, have been found to be associated with birth asphyxia in a few studies. For example, unspecific maternal fever, irrespective of cause and placental malaria, were identified as risk factors for birth asphyxia, and related deaths [14, 18, 19]. Furthermore, it is hypothesized that there are interactions between infections and other complications of labour, that cause compromise of placental gaseous exchange, leading to fetal hypoxia and then birth asphyxia [13]. Intrapartum complications such as pre-eclampsia, obstructed labour, prolonged labour, malpresentations, induction of labour, and having meconium-stained amniotic fluid, have been associated with birth asphyxia in many studies in Africa, and beyond [20–22].

We hypothesized that in addition to the common intrapartum risk factors for birth asphyxia, maternal infections such as; malaria, syphilis and other bacterial infections, and socio-demographic factors, play an important role in this study setting. Therefore, we aimed to determine the prevalence and to describe the contextual factors associated with birth asphyxia in a post conflict region of Uganda, in order to support planning for targeted interventions.

## Methods

### Study setting

The study was conducted at Gulu Regional Referral Hospital (Gulu RRH), and St. Mary’s Hospital-Lacor, in the Acholi region, Northern Uganda. Gulu RRH is a public Regional Referral facility, with a 347-bed capacity. During the study period, it had a maternity unit of 6 delivery beds, and around 4,400 births in a year. St. Mary’s Hospital-Lacor is a large private not for profit facility at the level of a referral hospital, with a 482-bed capacity. The maternity unit had 8 delivery beds, and an average of 6000 births annually. Both hospitals have the capacity to perform caesarean sections and offer emergency obstetric and newborn care interventions. Preference for where to seek care depends on the referring facility, and the woman’s individual ability to pay user charges at the private facility. These facilities receive referrals from primary care facilities within the neighboring districts, and beyond. These districts include; Nwoya, Amuru, Kitgum, Lamwo, Pader and Omoro.

### Study design and recruitment period

A cross-sectional study design was used. Data was collected from March 2018 to March 2019 in Gulu RRH, and from November 2018 to March 2019 in St. Mary’s Hospital Lacor.

### Study participants and eligibility

Women in established labour, who delivered at one of the two hospitals during the study periods, were included. We recruited women in the active phase of the first stage of labour with a cervical dilatation of ≥ 4 cm. Women who came in the second stage of labour were not invited to participate in the study, since it would be difficult to obtain consent. Women who; 1) were critically ill, for example those with eclampsia, and unable to respond to the questions, 2) were less than 37 weeks of gestation, 3) had multiple pregnancy or 4) had stillbirths were excluded.

### Sample size

To determine the factors associated with birth asphyxia, we calculated the sample size, using the Kelsey’s formula [23]. A sample size of 3,067 participants was calculated, assuming a 10% non-response rate, and a 95% confidence interval with 80% power. We used findings from a study in Cameroon, 22% of infants born to women with malaria, were found to have birth asphyxia, compared to 16% among those without [15].

### Data collection procedure

Data collection was done by six trained research assistants, who were nurses/midwives. The training of the research assistants included both theoretical and practical aspects of screening participants for eligibility, obtaining informed consent, drawing blood, determining the Apgar score, and completing the questionnaires. Potential study participants were consecutively informed about the study, by the midwife on duty. Thereafter, informed consent was obtained by the research assistants, to collect maternal blood samples for complete blood count and malaria, at admission; and cord artery blood sample collection at birth [24]. Furthermore, written informed consent was obtained from the participants after giving birth, to ensure that the information provided was clearly understood, before filling in the questionnaire.

Data was collected using paper-based questionnaires. Sections in the questionnaire included: socio-demographic characteristics, antenatal history, intrapartum events including laboratory tests and newborn characteristics. The socio-demographic characteristics and antenatal care history were obtained from the mother, while intrapartum events were abstracted from the mother’s maternity record. At birth, the research assistants were present, to score the newborn, using the Apgar scoring sheet.

### Sample collection and laboratory tests

Two milliliters (2 ml) of venous blood were collected from all mothers. The blood was stored in EDTA vacutainers, and analyzed for a complete blood count and Rapid Plasma Reagin (RPR) test, for syphilis. Peripheral maternal blood was also taken by a finger prick, and placental blood was taken from the vein, to make a thick malaria smear on a slide. All samples were tested at MBN, a clinical laboratory that is certified by HuQAS External Quality Assessment Services, situated within a 10-km radius of the study hospitals. Samples were picked daily, and transported to the laboratory for analysis. Results were returned to the research assistants, who included the findings in the questionnaire. Participants who were positive for any of the infections were treated, according to hospital protocols. In case the results returned after the mother’s discharge, she was contacted by phone, and asked to seek care from a nearby health facility.

### Study variables

#### Outcome variable

The main outcome of this study was birth asphyxia, defined as a 5-min Apgar score, less than 7 [2]. Apgar score is an acceptable method of determining birth asphyxia, especially in low resource settings where sophisticated measurements such as blood gas analysis are not readily available. The decision to use the Apgar score, was based on similar previous studies, in addition to the American Association of Pediatricians and American College of Obstetricians and Gynecologists’ guidelines that consider a score of < 7 at 5 min as non-reassuring [25].

#### Independent variables

These included socio-demographic and care seeking characteristics, maternal infections, intrapartum complications and newborn characteristics. Socio-demographic characteristics were: maternal age, educational level, marital status, employment and wealth quintiles. Age was collected in completed years from the mother’s report. A mother was considered to be employed, if she worked for money either at a personal business or at another person’s venture. Women who reported to be housewives or stayed at home, were considered unemployed. Level of education was defined according to the Ugandan system, where the first seven years of education, are referred to as primary level, the next six years are referred to as secondary level and thereafter, tertiary level. Wealth quintiles were computed, using principal component analysis [26] from indicators such as; home ownership of a car, a bicycle, a cupboard, a flask and standard of home including; having electricity, and the nature of the house in which a participant was living at the time of data collection. The household wealth indicators used in this study were adopted from a questionnaire for the Uganda demographic health survey of 2016 [4]. Parity indicated the number of births in which the pregnancy was carried to 28 weeks or more; whether the newborn had been alive or dead). Care seeking characteristics included: antenatal attendance, hospital of birth, distance from home to the hospital, taking intermittent presumptive treatment of malaria and fever during pregnancy. Maternal infections included; malaria, syphilis, HIV serostatus and the white blood cell count. Malaria infection was determined by a positive smear from both peripheral, and placental blood. A complete blood count was used to determine the hemoglobin level, and white blood cell count (WBC) of the mother. The hemoglobin levels were obtained as a continuous variable and categorized as low or anemia if < 11 g/dl or normal if ≥ 11 g/dl [27]. A WBC of more than 10 × 10^3^cells/mm^3^ was considered high as per the laboratory reference ranges. Syphilis was identified using the Rapid Plasma Reagin test. We did not conduct HIV testing, but used the results from the antenatal or maternity records of the mother. Information regarding complications during labour such as; obstructed labour, malpresentation, malposition, induction of labour and augmentation of labour, were reviewed from the maternity case records as per the diagnosis of the attending health care provider. Fetal distress was considered when the fetal heart rate at any time during labour was < 120 or > 160 beats per minute [28]. The duration of labour was determined from the onset of regular contractions to the birth of the baby. Any duration of > 18 h was regarded as prolonged labour. Mode of birth was categorized into vaginal birth, cesarean section and assisted vaginal delivery by vacuum extraction. Birth weight was categorized as; low birth weight (< 2500 g), normal (2500-3999 g) and large infants (≥ 4000 g) [29].

### Data management

Questionnaires were checked regularly for completeness by the site coordinator. Attempts were made to obtain all missing information, before the participant left the facility. To ensure privacy, questionnaires were kept securely in a locked cabinet, only accessible to the study team. Data were entered into Epidata version 3.1, and exported to Stata version 15 for cleaning and analysis [30].

### Data analysis

Descriptive statistics were used to summarize the maternal and newborn characteristics. The continuous variables were summarized using the median (IQR) because the data was not normally distributed. Categorical data were summarized using frequencies and percentages. Birth asphyxia was measured as a dichotomous variable and its prevalence described as a percentage. Bi-variable analysis was carried out to determine if a relationship existed between birth asphyxia and each of the independent variables. To determine factors independently associated with birth asphyxia, a multivariable logistic regression analysis was performed. All variables with *p-values* less than 0.05 at bivariable analysis were included in the multivariable regression analysis. Other important factors that had previously been associated with birth asphyxia such as placental malaria, and those with a potential relationship based on scientific plausibility were included in the regression analysis model. Fetal distress was not included in the model because it occurs along the causal pathway for birth asphyxia [31, 32]. Adjusted odds ratios (aOR) with their 95% confidence intervals (CI) were used to determine factors that were independently associated with birth asphyxia, and a *p*-value less than 0.05, was considered statistically significant. To overcome multi-collinearity, we run correlational statistics for all included factors, and removed one of the factors if a moderate to strong correlation (*r* ≥ *to* + *or—0.6*) existed between two variables [33]. For example, there was a high (77.8%) correlation between parity and maternal age, therefore, as recommended by Ranganathan, Pramesh, Aggarwal [34], parity was not included in the model. The final model included: age of the mother, employment status, wealth quintiles, hemoglobin levels, distance from hospital, place of birth, placental malaria, syphilis, white blood cell count, history of fever during pregnancy, referral status, labour induction/augmentation, malpresentation/malposition, duration of labour more than 18 h, obstructed labour, premature rupture of membranes, mode of birth, birth weight, and sex of the newborn. The Hosmer–Lemeshow goodness-of-fit test was done and the *p*-value was found to be 0.248 indicating that the model properly fitted the variables.

### Ethical considerations

Ethical clearance was obtained from Makerere University School of Health Sciences Research and Ethics committee (SHSREC 2017–051). All women were informed about the study on admission to the labour ward by the attending midwife, and invited to participate in the study. Those who were eligible to participate were given additional information by the trained research assistants. Verbal informed consent was obtained from women with strong contractions at admission, while those in early labour provided written informed consent in order to take blood samples and perform the Apgar scoring at birth. Further informed consent was sought after delivery, and all participants provided their written consent, before the study questionnaire was completed. Participants were informed of their right to participate voluntarily and that they could withdraw at any point during the study without affecting the care provided by the hospital. The study was carried out following the ethical principles in the Declaration of Helsinki.

## Results

**Fig. 1:**
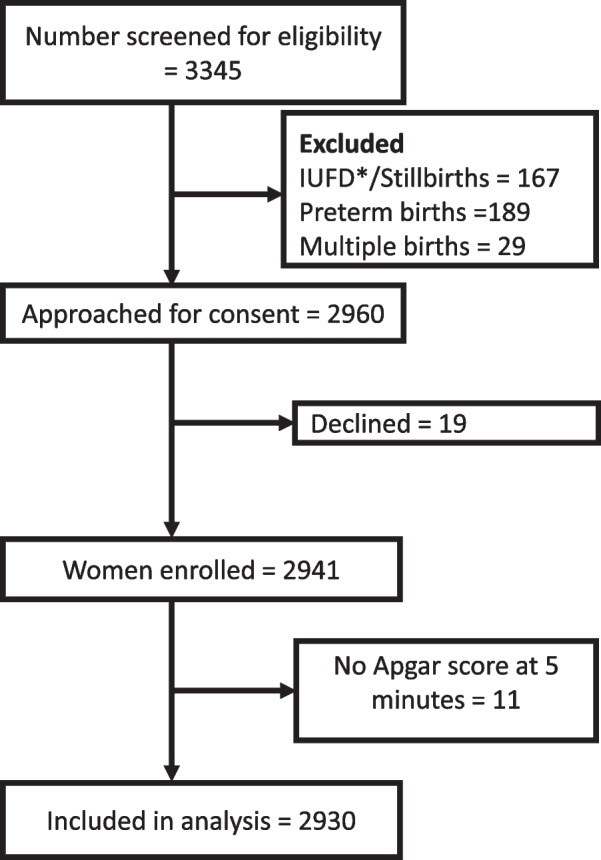
Flow chart showing screening and enrollment of participants

During the study period, 3,345 women were screened for eligibility to participate in the study, 2,941 were enrolled, and 2,930 were included in the analysis. The flow chart (Fig. 1) below shows the screening and enrolment process.

### Socio-demographic characteristics of mothers and newborns

The median age of the women was 23 (IQR: 20–28) years, and nearly a quarter of them, 721/2930 (24.7%) were 19 years or younger. The majority of women 1788/2924 (61.1%), were of low education status with primary or no formal education. Most of the women were unemployed (74.3%), and married or cohabiting (94.8%). The commonest mode of birth was vaginal (82.0%), while a few mothers required assisted delivery by vacuum extraction (1.4%). The median birth weight for the newborns was 3150 (IQR: 2850–3500) grams, and 83.6% were vaginal births. Detailed socio-demographic characteristics are shown in Table 1.

**Table 1:** Description of Socio-demographic characteristics of mothers and their newborns

Characteristics	Frequency (*N* = 2930)	Percentage (%)
**Maternal age in completed years**^**a**^		
≤ 19	721	24.7
20 +	2196	75.3
**Mother’s level of education**^**b**^		
None/Primary	1788	61.1
Secondary	923	31.6
Tertiary	213	7.3
**Husband’s level of education**^**c**^		
None/Primary	1102	37.9
Secondary	1368	47.0
Tertiary	439	15.1
**Employment status**^**d**^		
Unemployed	2174	74.3
Employed	753	25.1
**Wealth quintiles**^**e**^		
First (Poorest)	593	20.8
Second to Fifth	2254	79.2
**Marital status**^**f**^		
Single/divorced	148	5.2
Married/Cohabiting	2723	94.8
**Mode of birth**^**g**^		
Spontaneous vaginal birth	2376	82.0
Cesarean section	474	16.4
Vacuum extraction	47	1.6
**Sex of the newborn**^**h**^		
Male	1487	51.4
Female	1404	48.6
**Birth weight (grams)**^**i**^		
< 2500	115	4.0
2500 – 3999	2665	92.0
4000 +	115	4.0

Missing observations: ^a^13 missing, ^b^6 missing, ^c^21 missing, ^d^3 missing, ^e^83 missing, ^f^59 missing, ^g^33 missing, ^h^39 missing, ^i^35 missing

### Prevalence of birth asphyxia

The prevalence of birth asphyxia (Apgar score < 7 at five minutes) was 154/2930 [5.3% (95%CI: 4.5- 6.1)]. At one minute, 476/2928[16.3% (95% CI: 14.9–17.6)] newborns had an Apgar score of < 7. One in 10 newborns with birth asphyxia had very low Apgar scores ≤ 3 at 5 min.

### Socio-demographic, maternal and care seeking factors associated with birth asphyxia

A significant number of women, 1186/2928 (40.5%) were having their first birth. Nearly half of all participants 1399/2832 (49.4%) came from a distance within 5 Kms radius of the study sites and a higher proportion of women 79/923 (53.0%) with birth asphyxia came from a distance of more than 10 km. The proportion of women with hemoglobin levels < 11.0 g/dl indicative of anemia, was 18.2%. The majority 2917/2926 (99.7%) of women attended antenatal care visits with 57.3% of them completing four visits. Several socio-demographic characteristics were highly associated with birth asphyxia. The bivariable analysis showed that age ≤ 19 years, primiparity, mother’s educational level, being employed, and being in the lowest wealth quintile were significantly associated with birth asphyxia. Distance from home to hospital, place of birth, and having had fever during pregnancy, were also significantly associated with higher odds of birth asphyxia. Details are shown in Table 2.

**Table 2:** Birth asphyxia by socio-demographic, maternal and care-seeking characteristics

**Characteristics**	**Birth Asphyxia** *n* = 154	**No birth Asphyxia** *n* = 2776	**Total** ***N* = 2930**	**cOR (95%CI)**	***P*-value**
**Sociodemographic characteristics**					
**Maternal age (years)**					
≤ 19	68(44.2)	653(23.6)	721(24.7)	2.55(1.84–3.55)	< 0.001
20 +	86 (55.8)	2110(76.4)	2196(75.3)	1	
**Parity**^*π*^					
Primipara	86(56.2)	1100(39.6)	1186(40.5)	1.95(1.41–2.71)	< 0.001
Multipara	67(43.8)	1675(60.4)	1742(59.5)	1	
**Mother’s educational level (years)**					
None/Primary	111(72.6)	1677(60.5)	1788(61.1)	1.63(1.11–2.40)	0.013
Secondary	36(23.5)	887(32.0)	923(31.6)	1	
Tertiary	6(3.9)	207(7.5)	213(7.3)	0.71(0.30–1.71)	0.452
**Employment status**					
Unemployed	137(89.5)	2037(73.4)	2174(74.3)	1	
Employed	16(10.5)	737(26.6)	753(25.7)	0.32(0.19–0.54)	< 0.001
**Wealth quintiles**					
First (Lowest)	53(35.6)	540(20.8)	593(20.8)	2.21(1.56–3.13)	< 0.001
Second to Fifth	96(64.4)	2158(80.0)	2254(79.2)	1	
**Maternal characteristics**					
**Hemoglobin levels** **(g/dl)** ^*∞*^					
Low HB (< 11.0 g/dl)	36(23.7)	490(17.9)	526(18.2)	1.42(0.97–2.09)	0.074
Normal (≥ 11.0 g/dl)	116(76.3)	2247(82.1)	2363(81.8)	1	
**Care seeking characteristics**					
**Distance from home to hospital** **(Km)** ^*γ*^					
0–5 km	40(26.9)	1359(50.6)	1399(49.4)	1	
5.1–10 km	30(20.1)	480(17.9)	510(18.0)	2.12(1.31–3.45)	0.002
More than 10 km	79(53.0)	844(31.5)	923(32.6)	3.18(2.15–4.70)	< 0.001
**Number of antenatal care visits**^β^					
0–3	63(41.2)	1180(42.7)	1243(42.7)	0.94(0.67–1.30)	0.701
4 +	90(58.8)	1580(57.3)	1670(57.3)	1	
**Hospital**					
Public	58(37.7)	1407(50.7)	1465(50.0)	1	
Private	96(62.3)	1369(49.3)	1465(50.0)	1.70(1.22–2.38)	0.002
**Took IPT during pregnancy**^**£**^	138(90.2)	2508(90.7)	2646(90.7)	0.95(0.55–1.64)	0.844
**History of fever during pregnancy**^**k**^	88(57.9)	1160(42.1)	1248(42.9)	1.89(1.36–2.64)	< 0.001

cOR means crude odds ratio. Missing data: ^γ^ missing in 98 observations, ^π^missing in 2 observations, ^∞^missing 41 observations, ^β^missing 17 observations, ^£^missing 11 observations, ^k^ missing 21 observations

### Intrapartum infections, complications and newborn characteristics associated with birth asphyxia

Malaria infection during labour was higher in placental 61/2893 (2.1%), than in the peripheral blood samples 38/2893 (1.3%). Significantly, more 129/150 (86.0%) women with a high WBC count had newborns with birth asphyxia. In this population, the prevalence of HIV infection was 9.9%. There was no significant association between; placental malaria, syphilis, HIV, and birth asphyxia.

Nearly a quarter 427/2921 (14.6%) of the women were referred from other health facilities and 38.6% of these had newborns with birth asphyxia. The commonest complication reported was prolonged labour 899/2916 (30.8%). Fetal distress was found among 8.9% of women and of these, 63.2% gave birth to newborns with birth asphyxia. At bivariable analysis, all maternal complications during labour including: fetal distress, obstructed labour, prolonged labour, induction/augmentation of labour, mal-presentation/malposition, and premature rupture of membranes, were associated with birth asphyxia. In addition, newborn characteristics such as low birth weight, male sex, assisted vaginal birth by vacuum extraction, were associated with birth asphyxia. Detailed findings are displayed in Table 3.

**Table 3:** Bivariate analysis of birth asphyxia by intrapartum infections, complications, birth and newborn characteristics

**Characteristic**	**Birth Asphyxia** *n* = 154	**No birth Asphyxia** *n* = 2776	**Total** ***N* = 2930**	**Crude OR (95%CI)**	***P*-value**
**Intrapartum infections**					
Positive peripheral malaria^a^	1(0.6)	37(1.4)	38(1.3)	0.47(0.64–3.47)	0.461
Positive placental malaria^a^	5(3.3)	56(2.0)	61(2.1)	1.62(0.64–4.10)	0.310
Positive syphilis (RPR) test ^b^	9(5.9)	102(3.7)	111(3.8)	1.61(0.80–3.25)	0.181
Positive HIV status^c^	13(8.8)	256(9.9)	269(9.9)	0.88(0.49–1.58)	0.665
High WBC(> 10 × 10^3^/mm^3^) ^d^	129(86.0)	1956(71.9)	2085(72.7)	2.39(1.50–3.83)	< 0.001
**Intrapartum complications**^e^					
Referral status	59(38.6)	368(13.3)	427(14.6)	4.09(2.90–5.77)	< 0.001
Prolonged > 18 h	89 (58.2)	810(29.3)	899(30.8)	3.35(2.41–4.67)	< 0.001
Obstructed labor	32(20.8)	139(5.0)	171(5.9)	4.96(3.25–7.59)	< 0.001
Labor induction/augmentation	35(22.7)	187(6.8)	222(7.6)	4.06(2.71–6.10)	< 0.001
Premature rupture of membranes	11(7.1)	55(2.0)	66(2.3)	3.80(1.94–7.41)	< 0.001
Mal-presentation/ position	20(13.0)	72(2.6)	92(3.1)	5.60(3.31–9.46)	< 0.001
Fetal distress	96(63.2)	164(5.9)	260(8.9)	27.18(18.86–39.17)	< 0.001
**Birth and newborn characteristics**					
**Mode of birth**					
Spontaneous vaginal birth	99(65.6)	2277(82.9)	2376(82.0)	1	
Cesarean section	39(25.8)	435(15.8)	474(16.4)	2.06(1.40–3.03)	< 0.001
Assisted vaginal birth	13(8.6)	34(1.2)	47(1.6)	8.79(4.50–17.19)	< 0.001
**Sex of the newborn**					
Male	99(65.1)	1388(50.7)	1487(51.4)	1.82(1.29–2.56)	0.001
Female	53(34.9)	1351(49.3)	1404(48.6)	1	
**Birth weight**					
< 2500	12(8.0)	103(3.8)	115(4.0)	2.22(1.19–4.13)	0.012
2500—3999	133(89.3)	2532(92.2)	2665(92.0)	1	
4000 +	4(2.7)	111(4.0)	115(4.0)	0.69(0.25–1.89)	0.466

^a^ Variable missing in 37 observations

^b^missing in 40 observations

^c^variable missing 208 observations

^d^missing in 60 observations

^e^variables missing observations less than 15

### Multivariable analysis to identify factors independently associated with birth Asphyxia

At multivariable analysis, the following factors were significantly associated with birth asphyxia: i) young maternal age (aOR1.92, 95%CI: 1.27–2.91), ii) syphilis infection (aOR 2.45, 95%CI: 1.08–5.57), iii) a high WBC count (aOR 2.26, 95%CI: 1.26–4.06), iv) referral (aOR1.75, 95%CI: 1.10–2.79), v) induction/augmentation of labour (aOR 2.70, 95%CI: 1.62–4.50), vi) prolonged labour (aOR1.88, 95%CI: 1.25–2.83), vii) obstructed labour (aOR 3.40, 95%CI: 1.70–6.83), viii) malpresentation/malposition (aOR 3.00, 95%CI: 1.44–6.27) and, ix) assisted vaginal delivery (aOR 5.54, 95%CI: 2.30–13.30) had higher odds of getting newborns with birth asphyxia. Women who were employed were 57% less likely to give birth to newborns with birth asphyxia. Newborn factors associated with birth asphyxia included being male (aOR 1.92, 95%CI: 1.28–2.88) and having a low birth weight (aOR 2.20, 95%CI: 1.07- 4.50). Details are presented in Table 4.

**Table 4:** Multivariable analysis of factors associated with Birth Asphyxia

Characteristic	Crude Odds ratios (95% CI)	*P*-values	Adjusted Odds ratios (95%CI)	*P*-values
**Socio-demographic characteristics**				
Maternal age (years)				
≤ 19	2.55(1.84–3.55)	** < 0.001**	1.92(1.27–2.91)	**0.002**
20 +	1		1	
Education level				
None/Primary	1.63(1.11–2.40)	**0.013**	0.93(0.57–1.52)	0.771
Secondary	1		1	
Tertiary	0.71(0.30–1.71)	0.452	1.45(0.55–3.81)	0446
Employed	0.32(0.19–0.54)	** < 0.001**	0.43(0.22–0.83)	**0.013**
Wealth quintiles				
First (poorest)	2.21(1.56–3.13)	** < 0.001**	1.45(0.93–2.26)	0.100
Second to Fifth	1		1	
Hemoglobin levels (g/dl)				
Low HB (< 11.0 g/dl)	1.42(0.97–2.09)	0.074	1.29(0.81–2.05)	0.288
Normal (≥ 11.0 g/dl)	1		1	
**Care seeking characteristics**				
Distance from home to hospital				
0–5.0 km	1		1	
5.1–10.0 km	2.12(1.31–3.45)	**0.002**	1.41(0.79–5.50)	0.248
More than 10.0 km	3.18(2.15–4.70)	** < 0.001**	1.30(0.77–2.20)	0.330
Hospital of birth				
Public	1		1	
Private	1.70(1.22–2.38)	**0.002**	1.09(0.70–1.70)	0.711
History of fever in pregnancy	1.89(1.36–2.64)	** < 0.001**	1.36(0.92–2.01)	0.127
**Maternal infections in labour**				
Placental malaria	1.62(0.64–4.10)	0.310	0.33(0.07–1.45)	0.139
Syphilis infection	1.61(0.80–3.25)	0.181	2.45(1.08–5.57)	**0.032**
High WBC count	2.39(1.50–3.83)	** < 0.001**	2.26(1.26–4.06)	**0.006**
**Intrapartum, birth & newborn factors**				
Referral status	4.09(2.90–5.77)	** < 0.001**	1.75(1.10–2.79)	**0.018**
Prolonged labour > 18 h	3.35(2.41–4.67)	** < 0.001**	1.88(1.25–2.83)	**0.002**
Obstructed labor	4.96(3.25–7.59)	** < 0.001**	3.40(1.70–6.83)	**0.001**
Labor induction/augmentation	4.06(2.71–6.10)	** < 0.001**	2.70(1.62–4.50)	** < 0.001**
Premature rupture of membranes	3.80(1.94–7.41)	** < 0.001**	1.67(0.47–2.89)	0.735
Mal-presentation/position	5.60(3.31–9.46)	** < 0.001**	3.00(1.44–6.27)	**0.003**
Mode of birth				
Spontaneous vaginal birth	1		1	
Cesarean section	2.06(1.40–3.03)	** < 0.001**	0.75(0.41–1.39)	0.363
Assisted vaginal birth	8.79(4.50–17.19)	** < 0.001**	5.54(2.30–13.30)	** < 0.001**
Sex of the newborn				
Male	1.82(1.29–2.56)	**0.001**	1.92(1.28–2.88)	**0.002**
Female	1		1	
Birth weight (in grams)				
< 2500	2.22(1.19–4.13)	**0.012**	2.20(1.07–4.50)	**0.031**
2500—3999	1		1	
4000 +	0.69(0.25–1.89)	0.466	0.92(0.30–2.81)	0.883

## Discussion

Our large cross-sectional study, at two hospitals in a post conflict area in Northern Uganda, indicated that one in 20 newborns had birth asphyxia. Factors independently associated with birth asphyxia were; young maternal age, syphilis infection, a high WBC count, referral, obstructed labour, prolonged labour, induction or augmentation of labour, malpresentation or malposition, assisted vaginal delivery, male newborns and low birth weight.

The prevalence of birth asphyxia at 5.3% is of major concern, considering that birth asphyxia causes more than a third of all newborn deaths in Uganda [35]. Also, majority of these newborns require specialised or intensive care, that is difficult to provide in the study setting, due to lack of supplies, equipment or even skilled personnel [36]. Although the prevalence of birth asphyxia in this study is higher, compared to the 2.8% reported in 2003 at a National Referral hospital in Uganda [37], it is lower than the rates reported by studies in Ethiopia [38–40]. A systematic review of literature on the prevalence of birth asphyxia in East and central Africa also found a much higher prevalence of 15.9% [41]. In this study, we excluded preterm newborns, which might explain the relatively lower prevalence of birth asphyxia compared to other studies in similar settings in Africa.

Young mothers < 19 years of age were twice more likely to give birth to newborns with asphyxia. Studies in other low-income countries have reported a similar association although with varying age cut-offs [42]. Poor birth outcomes among young mothers such as preterm birth and low birth weight have been described by Artiga and Hinton [43] although an association with birth asphyxia is not well documented. Maternal age is of interest in this study setting where most of the current mothers were born during the insurgency that lasted 20 years, and affected people’s livelihoods [44, 45]. Insurgency and conflict affects critical infrastructure leading to low income, poor health services and malnutrition [46]. In addition, the social and economic position of women which is usually low for young mothers; affects their health seeking behaviours, ability to access care in a timely manner, and might lead to obstetric complications that lead to birth asphyxia [47, 48]. It is therefore not surprising that employment reduced the odds of birth asphyxia by 57% among women who took part in this study.

There was a significant relationship between birth asphyxia and women with syphilis infection. Maternal syphilis infection in pregnancy has been shown in other studies to lead to adverse birth outcomes including; spontaneous abortions, preterm birth, stillbirth and newborns who are small for gestational age [49]. Important to note is that, in Uganda, routine screening for syphilis is done at the initial antenatal visit and treatment offered to those who are infected [28]. However, repeat screening is not routinely done and adherence to treatment is not known. This could have explained the high prevalence of syphilis 3.8% found in this setting where nearly all women attended antenatal care visits. These findings are similar to those in a study among postnatal mothers in Western Uganda [50].

In this study, a high WBC count was associated with birth asphyxia which could be a proxy indicator for bacterial or viral infections [51]. Although normally, WBC values increase during pregnancy due to maternal physiological stress [52], infections could be another reason for the observed high proportions in this population [53, 54]. Maternal WBC of more than 12,000 mm^3^ was found to predict chorioamnionitis among women with premature rupture of membranes [55]. Therefore, other maternal infections such as urinary tract infections and chorioamnionitis could have contributed to birth asphyxia.

We hypothesized that malaria would be one of the important predictive factors. However, there was no statistically significant relationship between placental malaria and birth asphyxia. Although normally, Northern Uganda is a high malaria endemic area, this study was done at a time following massive indoor residual spraying by the Ministry of Health [56], which explains the low prevalence. In addition, the majority of women (90.7%) had received intermittent presumptive treatment for malaria during pregnancy. In Uganda, it is a policy for every pregnant woman to receive at least two doses of prophylaxis for malaria using pyrimethamine-sulfadoxine combination.

As expected, there was a statistically significant association between birth asphyxia, and most intrapartum complications namely: prolonged labour, obstructed labour, induction or augmentation of labour, malpresentation/malposition, and assisted vaginal delivery. This is similar to findings in other studies in Africa and other low income countries [41, 57]. These complications, are likely to cause a compromise in blood flow to the fetus leading to hypoxic events that may progress to birth asphyxia if relief measures are not instituted [58]. The association with assisted vaginal delivery could be because it is done when there is prolonged second stage or in cases of fetal distress when there is need to expedite the delivery [59]. Fetal distress or non-reassuring fetal heart rate arises as a result of intrauterine hypoxia and acidosis which may persist after birth presenting as asphyxia [58]. Referral during labour is commonly done following complications with the fetus or the woman. Majority of these women tend to have adverse birth outcomes which may explain why in this study, referral was associated with birth asphyxia [60]. In case of complications, interventions such as restrictive use of oxytocin, careful monitoring of labour and adequate and timely interventions for breech presentation and obstructed labour including cesarean section, may reduce the likelihood of poor outcomes [61, 62]. However, due to the challenges of the health system in the setting, studies have shown that interventions such as emergency cesarean delivery are rarely done in a timely manner [36, 63]. Health care providers may also lack the skills to conduct vaginal breech birth. In addition, proper management of induction or augmentation may be difficult due to poor monitoring of fetal heart rate and uterine contractions [64]. A complex interplay of several related factors, ultimately culminate into a newborn with birth asphyxia. Interventions such as careful monitoring of labour, adequate and timely interventions for breech presentation and obstructed labour, including CS, may reduce the likelihood of poor outcomes [61, 62]. However, due to the challenges of the health system in the setting, interventions such as performing an emergency cesarean section are rarely done in a timely manner [36, 63]. Health care providers may also lack the skills and confidence to conduct vaginal breech birth as stated in a qualitative study among midwives in the UK [65]. Therefore, multifaceted interventions with input from the end users, may be necessary to mitigate such challenges [66].

Low birth weight and being male has been associated with poor health outcomes in many settings. It is therefore not surprising, that the risk of birth asphyxia was increased in the population. In a study among very premature infants, males were found to have poor neurological and respiratory outcomes, compared to their female counterparts [67]. In another study among low-birth-weight infants, males were more likely to have birth asphyxia at one and five minutes, as well as poor outcomes compared to females [68]. The biological reasons for the sex differences regarding outcomes has not been clearly described.

### Strengths and limitations

Our large and comprehensive study in two hospitals is one of the few studies reporting the prevalence of birth asphyxia in a post-conflict region. Beyond the common indicators of socio-demographic and obstetric factors, we included infections such as syphilis, malaria, and WBC; to investigate the possible associations with maternal infections and birth asphyxia. A major strength of our study is the rigorous determination of the newborn Apgar scores, which was done by trained research assistants, who were not part of the hospital care. While excluding preterm newborns can be seen as a weakness, we know that the Apgar score used in this study is less valid in this group [69]. We believe that excluding preterm newborns is a strength as it ensured homogeneity.

The study also had some limitations. First, there could have been reporting bias on variables that were collected using the participant’s report for example; gestational age, history of fever, events during pregnancy, and duration of labour. Secondly, since this was a cross-sectional study, we were not able to ascertain a causal relationship with birth asphyxia. Lastly, we also found a low prevalence of placental malaria, which may have reduced our power to make conclusions on its association with birth asphyxia.

## Conclusion

There prevalence of birth asphyxia in this study was 5.3% which is comparable to some studies in low-income settings. Being referred, young maternal age, infections, intrapartum complications, male and underweight newborns, were associated with birth asphyxia. There is need for special attention to pregnant teenagers and women who have been referred in labour. Women with other complications such as prolonged labour, obstructed labour and malpresentations or malpositions and a high risk of infection should be prioritized for fetal heart rate monitoring and timely interventions to prevent birth asphyxia. Re-screening of pregnant women, and appropriate management of syphilis and other infections along the continuum of care for women should be prioritized.

## Data Availability

The datasets generated and analyzed during the current study are not publicly available, as they were collected for a PhD degree, and no provisions were made to make them publicly available in terms of participants’ consent, but are available from the corresponding author, on reasonable request.

## Abbreviations

aOR: Adjusted odds ratio
CBC: Complete Blood Count
CI: Confidence Intervals
IQR: Interquartile Range
IUFD: Intrauterine Fetal Death
RPR: Rapid Plasma Regain
SHSREC: School of Health Sciences Research and Ethics Committee
WBC: White Blood Cell
WHO: World Health Organisation
